# Permselectivity and Ionic Conductivity Study of Na^+^ and Br^−^ Ions in Graphene Oxide-Based Membranes for Redox Flow Batteries

**DOI:** 10.3390/membranes13080695

**Published:** 2023-07-26

**Authors:** Raphael Flack, Anna Aixalà-Perelló, Alessandro Pedico, Kobby Saadi, Andrea Lamberti, David Zitoun

**Affiliations:** 1 Department of Chemistry, Institute for Nanotechnology and Advanced Materials (BINA), Bar Ilan University, Ramat Gan 590002, Israel; raphy.flack@gmail.com (R.F.); kobbysd@gmail.com (K.S.); 2 Dipartimento di Scienza Applicata e Tecnologia (DISAT), Politecnico di Torino, Corso Duca degli Abruzzi, 24, 10129 Torino, Italy; anna.aixala@polito.it (A.A.-P.); alessandro.pedico@polito.it (A.P.); andrea.lamberti@polito.it (A.L.); 3 Istituto Italiano di Tecnologia, Center for Sustainable Future Technologies, Via Livorno 60, 10140 Torino, Italy

**Keywords:** redox flow batteries, graphene oxide, permselectivity, proton exchange membrane, functionalization, nanostructured materials

## Abstract

Permselectivity of a membrane is central for the development of electrochemical energy storage devices with two redox couples, such as redox flow batteries (RFBs). In RFBs, Br_3_^−^/Br^−^ couple is often used as a catholyte which can cross over to the anolyte, limiting the battery’s lifetime. Naturally, the development of permselective membranes is essential to the success of RFBs since state-of-the-art perfluorosulfonic acid (PFSA) is too costly. This study investigates membranes of graphene oxide (GO), polyvinylpyrrolidone (PVP), and imidazole (Im) as binder and linker, respectively. The GO membranes are compared to a standard PFSA membrane in terms of ionic conductivity (Na^+^) and permselectivity (exclusion of Br^−^). The ionic conduction is evaluated from electrochemical impedance spectroscopy and the permselectivity from two-compartment diffusion cells in a four-electrode system. Our findings suggest that the GO membranes reach conductivity and permselectivity comparable with standard PFSA membranes.

## 1. Introduction

The redox flow battery (RFB) is an electrochemical energy storage device that uses two different redox couples in separate tanks. RFBs present an interesting energy storage system for long-duration energy storage (6–12 h and more) due to their decoupled power and capacity, owing to the fact that the electrolyte is stored in external tanks and is flowed through a central cell for energy conversion. This is unlike most battery systems where the electrolyte is contained within a sealed cell, intrinsically linking power and capacity. This makes RFBs highly adaptable to be combined with renewable energy, solar, and wind, which are intermittent by nature. There are several RFB chemistries based on the Br_3_^−^/Br^−^ redox couple as catholyte due to the fast kinetics of the couple and the abundance of the element [1]. Compared to other RFB chemistries, the fast kinetics result in high energy round-trip efficiency and power density while the abundance of the element brings down the levelized cost of storage (LCOS). For instance, commercial vanadium RFBs display a high LCOS, partly due to the cost of the vanadium electrolyte. Among the alternatives, the high-power H_2_/Br_2_ RFB has been extensively studied and has shown the crossover as the main Achilles heel of the technology [2,3,4,5,6]. Other chemistries, like Zn/Br_2_ and polysulfide/bromine, also suffer from the crossover of bromide species [7]. Ion exchange membranes (IEMs) play an essential role in facilitating the transport of specific ions while preventing unwanted crossing (catholyte species and water) [8,9,10]. The permselectivity of membranes is a measure of their ability to selectively allow the passage of counter-ions while rejecting co-ions. The permselectivity is the most important characteristic of IEMs, it ranges from 0 to 1, where 1 indicates perfect selectivity for counterions. In the case of RFB, the ratio between cationic and anionic conductivity should be maximized to enable a high coulombic and voltage efficiency while minimizing the self-discharge and capacity decrease [11,12,13].

Cation exchange membranes (CEMs) are of interest due to their ability to differentiate between cations and anions conduction through the material. Among them, proton exchange membranes (PEM) are the most popular for their use in fuel cells and are often used in RFBs as a readily available on-the-shelf pick. In renewable energy applications, energy efficiency plays a critical role in assessing the overall performance of the whole cell, which depends on the materials used [14,15,16]. An appropriate membrane should have high conductivity and high permselectivity. It can be exposed by the impedance measurement as the ionic resistance in Ohm.cm^2^, which is later normalized by the thickness of the membrane to obtain the ionic conductivity in S/cm [17]. If the high proton conductivity of PEM is usually an excellent indicator of the high cationic conductivity for other small cations (Li^+^ or Na^+^), permselectivity needs to be reassessed for each system.

Perfluorosulfonic acid (PFSA) membranes (commonly called Nafion) are usually used as PEM and have also been used for sodium transport, despite their high cost [18,19,20]. The spontaneous formation of hydrophilic and hydrophobic nanochannels in the PFSA has been shown to be instrumental for high cationic conductivity [21,22,23]. This discovery has fostered research on artificial nanostructures made of composite materials. To be sure, 2D material membranes have gained popularity in recent years due to the failure to progress with ionomer-type membranes [24,25,26,27]; 2D material membranes typically form by stacking 2D flakes, resulting in nanopores formed between the stacked sheets. These membranes have a high conductivity potential due to the density of pores. The selectivity of these membranes is typically imparted by the steric hindrance of the small interlayer spacing that forms the pores, although functional groups can be added to increase selectivity [28,29,30].

Carbon-based compounds with varied forms and physicochemical properties, including graphene oxide (GO), have been produced for over a century. GO membranes are made of dispersed GO flakes that are rearranged in parallel to form a stacked membrane. This creates a network of channels that act as precise molecular sieves [31,32]. These channels can be modified to allow precise control over the species that pass through and to transport small ions through them in a quick manner. The channel size and, hence, selectivity can be altered by physical compression, partial reduction of the fabricated GO membrane, and crosslinking the GO flakes [33,34]. These techniques offer the potential for the development of higher-quality membranes for redox flow batteries and other applications. According to A. Pedico et al. (2023), the improvement of the GO membranes can also be investigated by the process of fabrication [35].

In addition to electrochemical parameters, mechanical properties are also important for the performance of membranes used in electrochemical devices [15]. Properties such as power density, tensile strength, impact strength, flexural strength, hardness, and fracture toughness are essential for fabricating high-quality membranes that can withstand the operating conditions of a redox flow battery (RFB). Tensile strength is particularly important, and studies have been conducted to improve it by adding crosslinking to the membrane matrix [36]. Overall, the mechanical properties of membranes play a decisive role in ensuring the robustness and durability of the RFB system.

Remarkably, understanding sodium and bromide ions’ behavior and evolution through PFSA and modified (or unmodified) 2D materials, specifically with NaBr as the electrolyte, remains lacking in existing studies. This knowledge gap highlights the need to investigate and compare materials in terms of their ionic conductivity and permselectivity.

Therefore, our research aims to bridge this gap by conducting a comprehensive analysis. Initially, we will evaluate the permselectivity and ion conductivity of PFSA to gain insights into its strengths and weaknesses within this specific system. Subsequently, we will explore the transport of sodium and bromide ions in graphene oxide membranes modified with binders and linkers, such as polyvinylpyrrolidone (PVP) and imidazole (Im), respectively. Previous studies have indicated that PVP as a binder can enhance mechanical properties [35,37], while Im as a linker can intercalate into the interlayer spaces of the GO matrix, modifying the selectivity of the membrane pores [38,39].

By undertaking these investigations, we aim to better understand the ion transport characteristics through PFSA membranes and potential improvements in GO membranes. Additionally, we seek to establish a foundation for the comparative study of various materials in terms of their ionic conductivity and permselectivity.

## 2. Materials and Methods

### 2.1. Nafion 117 Membrane Activation Treatment

The commercial PFSA Nafion 117 sheet was cut into 4 cm × 4 cm squares and peeled off from the thin backing layer. The pre-cut squares were then soaked in water with 3% H_2_O_2_ (purchased from Carlo Erba Reagents, Evreux, France) for 1 h in lightly boiling (~80 °C). The membranes were rinsed in DI water and boiled again for 2 h in lightly boiling H_2_O. The activation was then done with 1 h of lightly boiling into 0.5 M H_2_SO_4_ (purchased from Carlo Erba Reagents, Evreux, France). The process was finished by finally doing 2–3× rinse in lightly boiling (80–90 °C) DI water before storing it in DI water.

### 2.2. GO Membrane Fabrication

**Figure 1:**
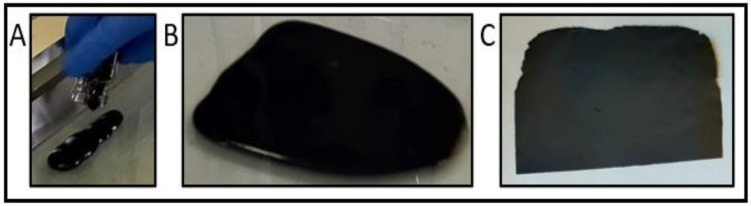
GO membrane slurry (**A**,**B**) and after being cast on the glass support (**C**).

GO gel (20 mg/mL) was purchased from Graphenea company and used for the fabrication of the membrane. Then, 5 g of GO gel was weighed to obtain 100 mg of GO in the final membrane, and the PVP (from Alfa Aesar, Thermo Fisher Scientific, Heysham, Lancashire, UK) and imidazole (from Alfa Aesar, Thermo Fisher Scientific, Heysham, Lancashire, UK) were weighed to be 10% in mass of the initial GO quantity and diluted into 1 mL miliQ water, respectively. To produce GO, GO-PVP, GO-imidazole, and GO-PVP-imidazole membranes, it was mixed accordingly with the wanted solutions and placed under stirring overnight. The next day, the solutions were cast on top of glass support (Figure 1A) and spread with a doctor’s blade at a support-to-blade distance of 1 mm (Figure 1B). The membrane was dried at room temperature for 12 to 48 h, and mechanically detached from the glass support, as seen in Figure 1C.

### 2.3. Characterization

High-resolution **scanning electron microscopy (HRSEM) images** were taken with a field emission Magellan 400 L HRSEM (FEI, Hillsboro, OR, USA), and the cross-section is determined by breaking the sample in liquid nitrogen and tilting the sample. The evolution of the crystallographic structure was determined by **XRD measurement**s, made on a Bruker AXS D8 advance, Cu Kα = 1.5418 Ǻ radiation (Mannheim, Germany). Bragg’s law was used to obtain the d-spacing value from the membranes. **Raman scattering measurements** were taken using a micro-Raman LabRam HR-800 (Horiba Jobin Yvon apparatus, Palaiseau, France), consisting of a single spectrograph equipped with a He-Ne laser (633 nm emission line) and with an optical microscope (Olympus, BX41). A long working distance (LWD) 80× objective, having a numerical aperture (NA) = 0.75 and yielding a spatial resolution of about 1 µm was used to focus the laser beam onto the sample surface in air at room temperature. **Infrared spectra** were taken neat on a Fourier-Transform Infra-Red (FTIR) spectroscope Nicolet iS10 (Thermo Scientific, Tewksbury, MA, USA). Only the significant peaks (medium intensity and greater) are listed.

### 2.4. Permselectivity and Ionic Conductivity Measurement

**Figure 2:**
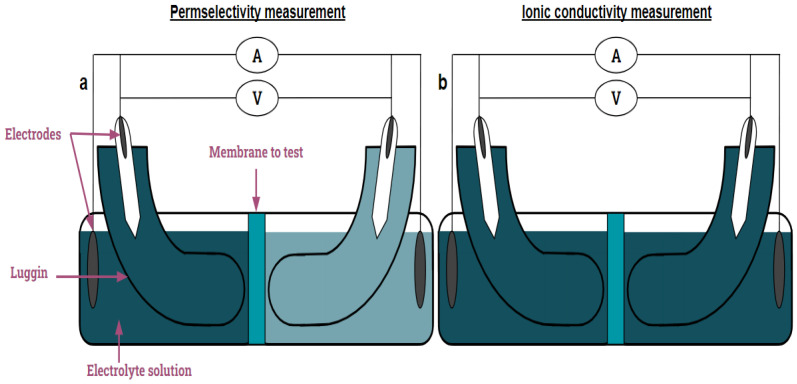
Scheme of the electrochemical measurements that will be pursued: (**a**) permselectivity; (**b**) ionic conductivity.

Two analyses were pursued in the same cell: (1) permselectivity and (2) ionic conductivity. Both experiments shared the cell, reference electrodes (RE, SE), and working and counter electrodes (WE, CE). Ag/AgCl reference electrodes were used as SE and RE. Carbon-based electrodes clamped with a titanium mesh were used as WE and CE. Nevertheless, the concentration of the electrolyte used was different, as seen in Figure 2: (a) permselectivity measurements require a concentration gradient (i.e., NaCl 0.1 M and 0.5 M), and (b) ionic conductivity measurement was pursued at the same concentration at each side of the membrane (i.e., NaCl 0.5 M).

In this research, NaBr electrolyte will mainly be used to obtain an understanding of the Na and Br ions’ behavior (conductivity and permselectivity) through the membrane’s materials.

## 3. Results

The chemical stability and mechanical endurance of the membranes have been previously reported [40]. In summary, the membranes are chemically stable in concentrated NaBr solutions and mechanically robust.

### 3.1. Electrochemical Characterization (Ionic Conductivity–Permselectivity)

**Figure 3:**
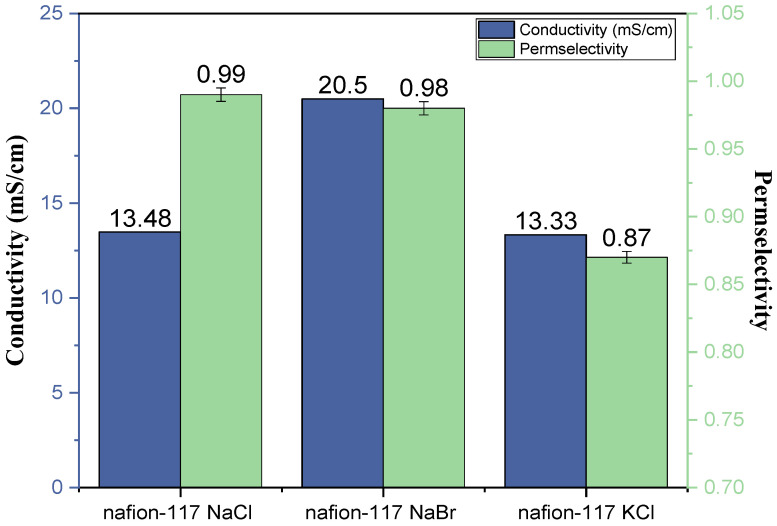
Ionic conductivity (blue) and permselectivity (green) results of PFSA (Nafion 117) in different electrolytes (NaCl, NaBr, KCl).

**Figure 4:**
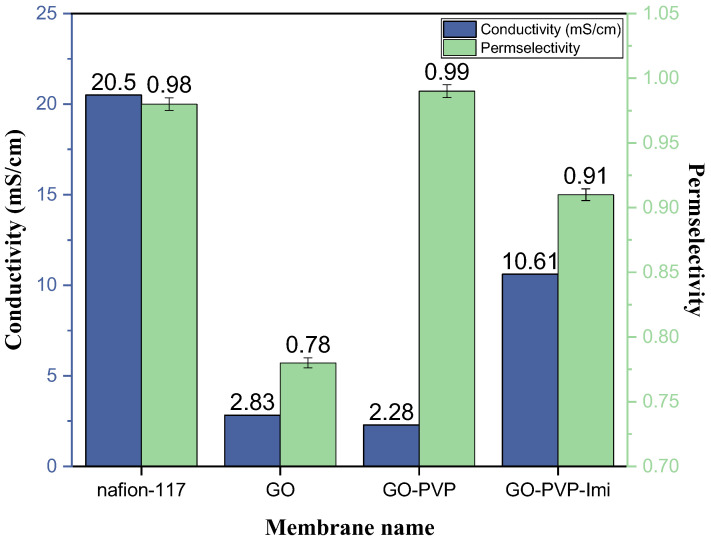
Co ionic conductivity (blue) and permselectivity (green) measurement on PFSA and different GO-based membranes in NaBr electrolyte.

The results are reported in two different forms. The main manuscript reports the ionic conductivity and the permselectivity for PFSA and GO membranes, in Figure 3 and Figure 4, respectively. The Supporting Information reports the ionic resistance and the membrane potential for all the membranes in the NaBr electrolyte (Figure S1). The membrane potential is one variable in the permselectivity calculation. For the measurement of PFSA (Figure 3), similar permselectivity can be observed between NaCl and NaBr electrolytes while the ionic conductivity is much improved by the presence of bromide ions compared to chloride ions. This highlights a partial crossover of the bromide, which globally increases the final conductivity observed. When the cation is replaced (from NaCl to KCl electrolyte), the ionic conductivity is conserved around 13 mS/cm, whereas the permselectivity is much impacted (0.99 for NaCl to 0.87 for KCl). Knowing the cation migration through the PFSA material is by the hopping mechanism, we can consider both cations without their hydration layer. Consequently, it appears the cation size affects the conductivity.

The initial results comparing graphene oxide to PFSA (Figure 4) reveal a lower initial ionic conductivity of 2.83 mS/cm for GO compared to 20.50 mS/cm for Nafion. Upon treatment with polyvinylpyrrolidone, the GO-PVP membrane exhibits an improved permselectivity, increasing from 0.78 to 0.99, which makes it comparable and even slightly higher than PFSA. Remarkably, the ionic conductivity of GO-PVP remains low compared to the initial GO. This highlights the efficacy of PVP in providing comprehensive stability and rigidity to GO membranes, thereby impeding the passage of anions.

After the introduction of imidazole to the GO-PVP membrane (forming GO-PVP-imidazole), the ionic conductivity increases to 10.61 mS/cm, albeit at the cost of a reduced permselectivity of 0.91. The incorporation of imidazole enhances the conductivity while compromising the permselectivity.

Considering that PVP initially supports the formation of a more stable matrix, and imidazole enhances the conductivity between the flakes of GO, these findings collectively demonstrate the simultaneous modulation and improvement of two key parameters, namely, ionic conductivity and permselectivity, in the 2D material. Although the results are not yet as favorable as those achieved with PFSA, they underscore the potential for enhancing both properties in tandem, while certainly reducing the cost of the membrane.

In another case, electrochemical impedance spectroscopy (EIS) and open-circuit voltage (OCV) were conducted in order to obtain the ionic resistance in Ohm.cm^2^ and the voltage in mV.

### 3.2. IR Spectroscopy

**Figure 5:**
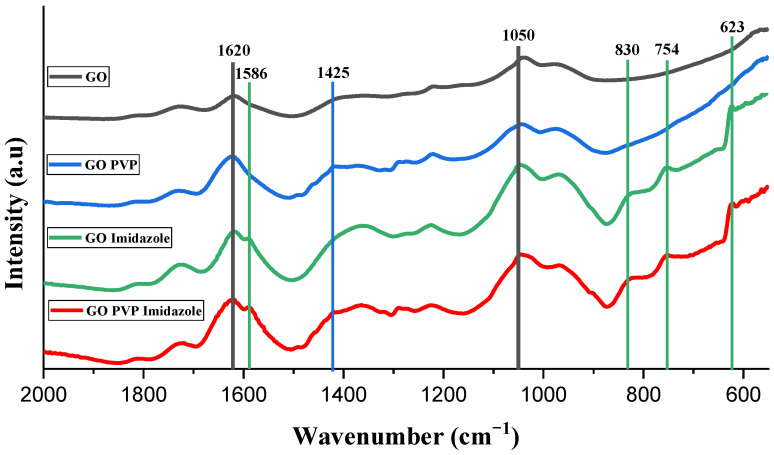
Absorbance IR spectrum of all the GO membranes (untreated and treated by PVP and/or imidazole).

Fourier transform infrared (FTIR) spectra of the different GO-based membranes with the different treatments (PVP and/or imidazole) are shown in Figure 5. From the initial pure GO membrane, the addition of PVP can be seen by the decrease of the band at 1050 cm^−1^, corresponding to the epoxy groups for the appearance of a C-N band at 1425 cm^−1^ as, in fact, PVP contains tertiary amine. For the treatment with imidazole, three bands appear between 600 and 900 cm^−1^, characteristic of N-H vibrations, and a shoulder at 1586 cm^−1^ appears (vibration N-H also). The band at 1620 cm^−1^ does not change so much as the C-N vibration band is getting at the same place as the C-O band, yet seeing this band not increasing suggests a replacement of the C-O by C-N and not the addition of the two vibrations.

### 3.3. Raman

**Figure 6:**
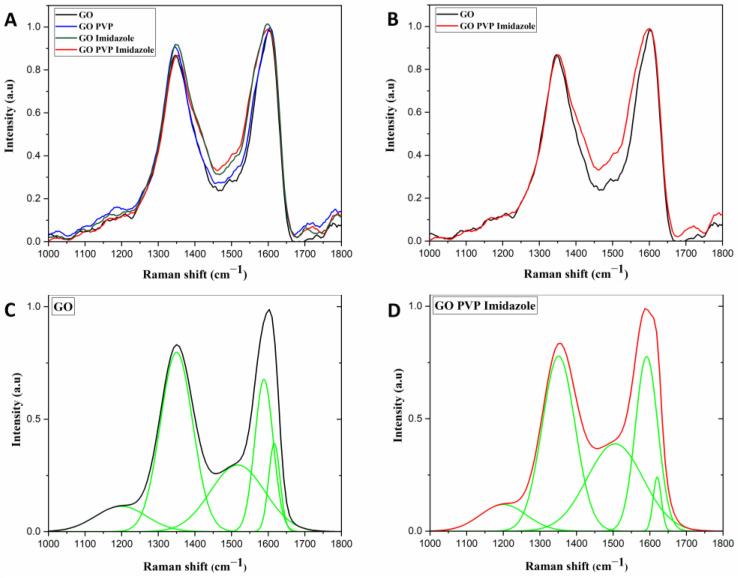
Raman of all the GO membranes, untreated and treated by PVP and/or imidazole in (**A**), comparison between pure GO and GO PVP Imidazole in (**B**), and the deconvolution of these two graphs in (**C**,**D**).

Raman was performed as another way to obtain information on the modifications induced by PVP and imidazole addition (Figure 6). It can be seen first in Figure 6A, there is no specific modification between GO and GO-PVP, whereas the two membranes containing imidazole have two shifts. When we look at Figure 6B–D, the two shifts appear to be one from 1525 to 1500 cm^−1^ for the D” band and one from 1400 to 1425 cm^−1^ for the D* band. Based on the research from Sergi Claramunt et al. (2015) [41], the D″ band decreases and D* increases following the diminution of oxygens in the GO. This would mean a reduction of the graphene oxide induced by the imidazole that may react covalently as expected between the sheets of GO, which brings an increase in the crystal ordering of the material.

Moreover, due to an initial fluorescence for GO [42,43] and PVP [44] and a stronger one when treated with imidazole, a quenching was done for 10 min before every measurement. Imidazole does not generate fluorescence in the wavelength window of analysis [45]. It can be noticed that the increase in the fluorescence when GO is treated with imidazole can show the creation of a covalent bond between the two components inducing a hyperchromic and bathochromic effect by the emission shifting to the red and increasing in intensity. To summarize, the PVP does not seem covalently attached as a binder is supposed to do when the imidazole is covalently attached at the location of the carboxylic and carbonyl functional group as expected for a linker.

### 3.4. XRD

**Figure 7:**
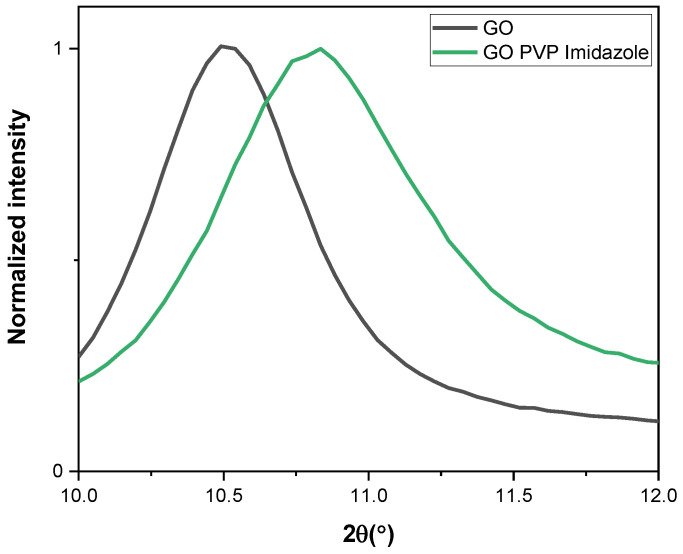
XRD of GO membranes (untreated and treated by PVP and/or imidazole).

XRD was used for a better understanding of the structure and order of the GO matrix (Figure 7). All GO-based membranes exhibit a specific pic around 10 degrees, characteristic of the interlayer distance between GO flakes [46]. This indicates that the treatments with PVP and Im do not affect the ordered structure of the stack.

Yet, interestingly, some differences can still be noticed through the modifications. The reflection corresponding to the interlayer spacing of GO is located at 10.49 degrees and for GO PVP Im at 10.83 degrees, meaning 8.43 to 8.16 A, respectively. The treatment of GO has led to a diminution of the interlayer space in the GO structures. On top of it, a wider reflection can be noticed after treatment, characteristic of less ordered material. To summarize, it seems a more structured membrane with a reduced interlayer space (made by the linker Im) displays an increased amorphous phase (induced by the binder PVP).

### 3.5. SEM

A cross-sectional analysis of the GO-based membrane using SEM was carried out to characterize the membrane thickness and its structure. It was observed (in Supplementary Materials Figure S2) that the membrane had an approximate thickness of 18.84 μm for GO, 15.15 μm for GO PVP, 13.38 μm for GO Imi, and 27.88 μm for GO PVP Imi. However, the morphology appeared to be influenced by the razor blade cut (even though it was carefully sectioned), making it challenging to clearly discern the lamellar structure. The impact of the cutting method is evident in the SEM images, where the lamellar arrangement is not readily distinguishable.

**Figure 8:**
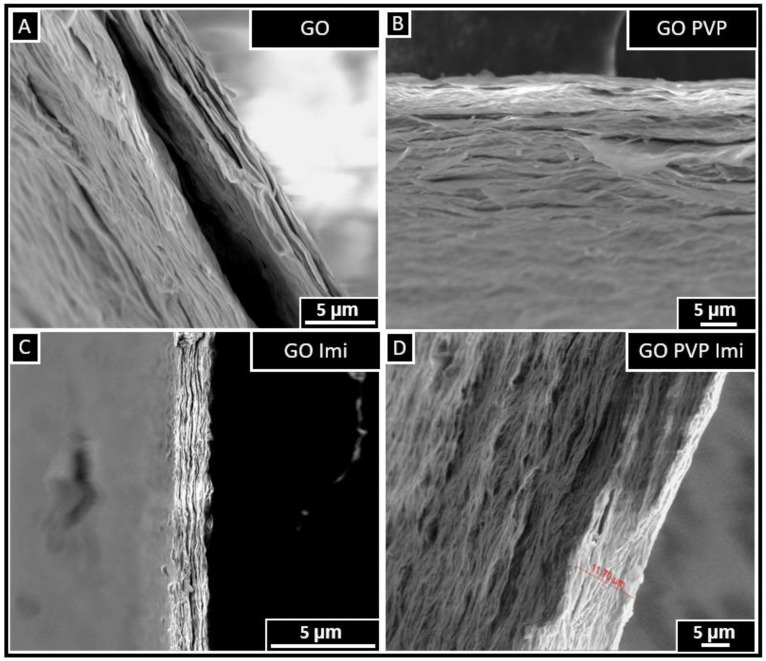
SEM images of the cross-section of GO-based membrane following freeze cutting: GO (**A**), GO PVP (**B**), GO imidazole (**C**), GO imidazole PVP (**D**).

Additional analyses were conducted on the cross-section again after a freeze cut to preserve the morphology of the membranes. Here, the lamellar structure of the membranes induced by the flakes of GO appeared clearer (Figure 8) for all of the membranes. It can even be seen as a crack between the layers on the GO picture due to the liquid nitrogen treatment.

## 4. Discussion

The results obtained from our study shed light on the important aspects of ionic conductivity and permselectivity in RFB membranes. The evaluation of PFSA revealed comparable permselectivity between NaCl and NaBr electrolytes, while the presence of bromide ions significantly improved the ionic conductivity compared to chloride ions. This observation indicates a partial crossover of bromide ions, leading to an overall increase in conductivity and yet, also highlighting the issue of self-discharge, voltage, and capacity decrease. However, when the cation was changed from sodium to potassium, the permselectivity was significantly reduced while the ionic conductivity remained relatively constant. This suggests that cation size influences conductivity in Nafion, where cation migration occurs through the hopping mechanism without considering the hydration layer.

In the initial comparison between GO and PFSA membranes, it was found that PFSA exhibited higher initial ionic conductivity compared to GO. However, after treating the GO membrane with polyvinylpyrrolidone (PVP), an improvement in permselectivity was observed, reaching a value comparable to that of PFSA (0.99). Notably, the ionic conductivity of GO-PVP remained unchanged compared to the initial GO membrane, highlighting the effectiveness of PVP in providing comprehensive stability, and rigidity to the GO membranes, thereby impeding the passage of anions. The introduction of imidazole to the GO-PVP membrane (GO-PVP-imidazole) resulted in an increase in ionic conductivity, albeit with a slight reduction in permselectivity. This incorporation of imidazole enhanced the conductivity between the GO flakes while compromising some of the permselectivity. The combined use of PVP and imidazole demonstrated the simultaneous modulation and improvement of ionic conductivity and permselectivity in the 2D material.

Although the results obtained with GO membranes modified with PVP and imidazole are not yet as favorable as those achieved with PFSA, they highlight the potential for enhancing both properties in tandem. This suggests that further optimization and refinement of the GO membrane structure and modification techniques may lead to even better performance. It is important to note that while PFSA has been extensively studied and widely used, our research addresses the need for exploring alternative materials like GO, which offer the potential for improved performance and lower cost.

## 5. Conclusions

RFBs require specific CEM to reach high ionic conductivity and high permselectivity with low-cost materials. The standard and pricy CEM, namely PFSA, shows high conductivity (20.5 mS/cm) and permselectivity (0.98) in NaBr electrolyte, one of the most used catholyte in RFB. In this report, we show that GO membrane approaches PFSA values with an additional linker and binder, respectively, imidazole (10.61 mS/cm) and PVP (permselectivity 0.99), while keeping the cost of raw materials very low. Overall, our findings contribute to the understanding of ion transport characteristics and the potential for enhancing the conductivity and permselectivity of GO membranes. These insights provide a basis for further research and the development of higher-quality membranes for redox flow batteries and other electrochemical devices. Future studies can explore additional modification techniques and investigate the trade-off between conductivity and permselectivity in different 2D materials to advance the field of RFB membranes and contribute to the development of more efficient and reliable energy storage systems.

## Data Availability

The data that support the findings of this study are available from the corresponding author, D.Z., upon reasonable request.

## Supplementary Materials

The following supporting information can be downloaded at: https://www.mdpi.com/article/10.3390/membranes13080695/s1, Figure S1: EIS (in Ohm.cm^2^) and OCV measurement on Nafion and GO-based membranes in NaBr electrolyte; Figure S2: SEM image of the cross-section for the pure GO membrane. Reference [47] is cited here.
