# Supplementary material for: Permselectivity and Ionic Conductivity Study of Na+ and Br− Ions in Graphene Oxide-Based Membranes for Redox Flow Batteries
Source: Membranes (Basel). 2023 Jul 26;13(8):695. doi: 10.3390/membranes13080695 (PMC10456580; doi:10.3390/membranes13080695)
Supplement: Supplementary file 1 [file membranes-13-00695-s001.zip › membranes-2488511-supplementary.pdf]

Supplementary Materials

Permselectivity and Ionic conductivity study of Na^+ and Br^- ions in Graphene Oxide - based membranes for Redox Flow Batteries

Raphael Flack [1], Anna Aixalà-Perelló [2], Alessandro Pedico [2], Kobby Saadi [1], Andrea Lamberti [2] and David Zitoun [1,*]

[1] Bar Ilan University, Department of Chemistry, Institute for Nanotechnology and Advanced Materials (BINA), 590002 Ramat Gan, Israel

[2] Politecnico di Torino, Dipartimento di Scienza Applicata e Tecnologia (DISAT), Corso Duca degli Abruzzi, 24, 10129 Torino, Italy

1. EIS and OCV

Electrochemical Impedance Spectroscopy (EIS) and Open-Circuit Voltage (OCV) were done in order to obtain the ionic resistance in Ohm.cm^2 and the voltage in mV.

After obtaining the thickness of the membranes by SEM in cross-section cut (Supplementary Materials S2), the thickness was divided by the ionic resistance to obtain the ionic conductivity in mS.cm.

For the Permselectivity, the Voltage from the experiment was compared to the theoretical one in the "apparent membrane permselectivity" formula from the article of Ryan S. Kingsbury (2018) [1].

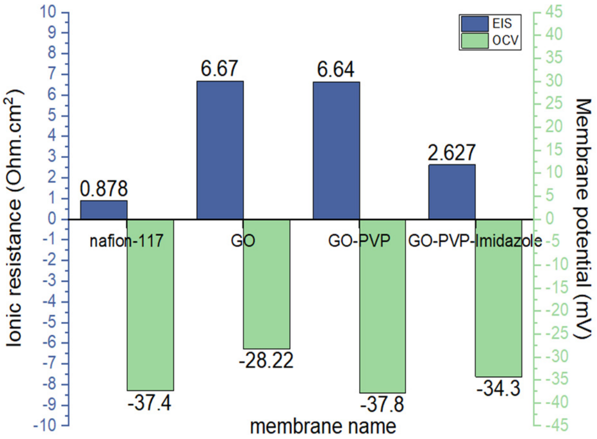

Figure S1. EIS (in Ohm.cm^2) and OCV measurement on Nafion and GO-based membranes in NaBr electrolyte.

2. SEM

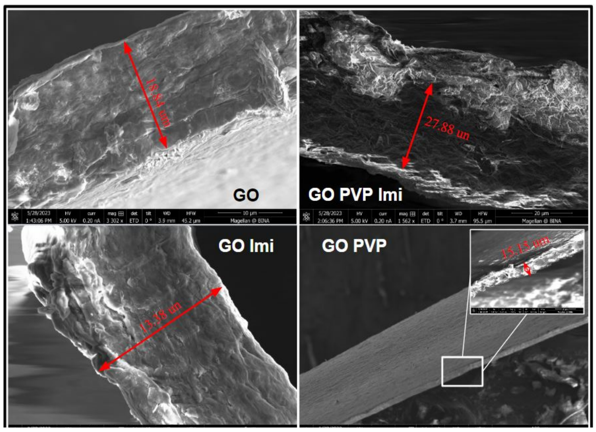

Figure S2. SEM image of the cross-section for the pure GO membrane.

References

1. Kingsbury, R. S.; Flotron, S.; Zhu, S.; Call, D. F.; Coronell, O. Junction Potentials Bias Measurements of Ion Exchange Membrane Permselectivity. Environ. Sci. Technol. **2018**, 52, 4929–4936.
